# The effect of liquid consistency on penetration-aspiration: a Bayesian analysis of two large datasets

**DOI:** 10.3389/fresc.2024.1337971

**Published:** 2024-02-23

**Authors:** James C. Borders, Catriona M. Steele

**Affiliations:** ^1^ Laboratory for the Study of Upper Airway Dysfunction, Department of Biobehavioral Sciences, Teachers College, Columbia University, New York, NY, United States; ^2^ Swallowing Rehabilitation Research Laboratory, KITE Research Institute—University Health Network, Toronto, ON, Canada; ^3^ Department of Speech-Language Pathology, Rehabilitation Sciences Institute, Temerty Faculty of Medicine, University of Toronto, Toronto, ON, Canada; ^4^ Canada Research Chair (Tier 1) in Swallowing and Food Oral Processing, Canada Research Chairs Secretariat, Ottawa, ON, Canada

**Keywords:** deglutition disorders, dysphagia, aspiration, videofluoroscopy, thickened liquids, consistency

## Abstract

**Introduction:**

Thickened liquids are commonly recommended to reduce the risk of penetration-aspiration. However, questions persist regarding the impact of bolus consistency on swallowing safety. The common practice of summarizing Penetration-Aspiration Scale (PAS) scores based on worst scores is a bias in prior analyses. The aim of this study was to examine the impact of liquid consistency on PAS scores using a Bayesian multilevel ordinal regression model approach, considering all scores across repeated bolus trials. A second aim was to determine whether PAS scores differed across thickener type within consistency.

**Methods:**

We analyzed two prior datasets (D1; D2). D1 involved 678 adults with suspected dysphagia (289 female; mean age 69 years, range 20-100). D2 involved 177 adults (94 female; mean age 54 years, range 21-85), of whom 106 were nominally healthy and 71 had suspected dysphagia. All participants underwent videofluoroscopy involving ≥3 boluses of 20% w/v thin liquid barium and of xanthan-gum thickened barium in mildly, moderately and extremely thick consistencies. D2 participants also swallowed trials of slightly thick liquid barium, and starch-thickened stimuli for each thickened consistency. Duplicate blinded rating yielded PAS scores per bolus, with discrepancies resolved by consensus. PAS ratings for a total of 8,185 and 3,407 boluses were available from D1 and D2, respectively. Bayesian models examined PAS patterns across consistencies. We defined meaningful differences as non-overlapping 95% credible intervals (CIs).

**Results:**

Across D1 and D2, penetration occurred on 10.87% of trials compared to sensate (0.68%) and silent aspiration (1.54%), with higher rates of penetration (13.47%) and aspiration (3.07%) on thin liquids. For D1, the probability of a PAS score > 2 was higher for thin liquids with weighted PAS scores of 1.57 (CI: 1.48, 1.66) versus mildly (1.26; CI: 1.2, 1.33), moderately (1.1; CI: 1.07, 1.13), and extremely thick liquids (1.04; CI: 1.02, 1.08). D2 results were similar. Weighted PAS scores did not meaningfully differ between thin and slightly thick liquids, or between starch and xanthan gum thickened liquids.

**Discussion:**

These results confirm that the probability of penetration-aspiration is greatest on thin liquids compared to thick liquids, with significant reductions in PAS severity emerging with mildly thick liquids.

## 1 Introduction

Thickened liquids are commonly recommended as an intervention for people with dysphagia in whom airway invasion (“penetration-aspiration”) of thin liquids is suspected or confirmed based on swallowing assessment. In the very first year of the *Dysphagia* journal, Coster & Schwarz introduced the concept of the “swallow safe bolus”, described as a bolus with properties that enable its “unimpeded passage through the food pathway without aspiration, choking, or retention in the pharynx” (1). Subsequently, Curran & Groher (2) described the inclusion and exclusion of foods and liquids with specific characteristics in the “aspiration risk reduction diet” in a Veterans' Affairs hospital in New York, including the thickening of liquids to three different degrees, which were labelled nectar consistency, honey consistency and pudding consistency. Despite recommendations in textbooks that thickened liquids should be considered as an intervention of last resort, and that other compensatory interventions should be considered first as techniques for reducing penetration-aspiration (3), the literature suggests that thickened liquids quickly became one of the most recommended interventions for dysphagia (4).

The most-commonly cited research study exploring the efficacy of thickened liquids for reducing penetration-aspiration was led by Logemann & Robbins, often referred to as “Protocol 201”. In the first phase of that study, a sample of 711 adults with diagnoses of Parkinson Disease and/or dementia were enrolled based on videofluoroscopic confirmation of at least one occurrence of aspiration across a series of three 3 ml boluses and three sips of thin liquid barium. These individuals were then asked to swallow up to 6 boluses each, including three 3 ml boluses and three sips of: (a) thin liquid using a chin-down posture; (b) nectar-like liquid barium; and (c) honey-like barium. The order of these intervention conditions was randomized across participants. The study results showed improved swallowing safety in 51% of participants, with the remaining 49% of participants continuing to aspirate in all three intervention conditions. Twenty-five percent of the sample showed improved swallowing safety with all three interventions, while 26% showed a best response, with the honey-like barium showing significantly lower aspiration rates compared to both the nectar-like barium and the chin-down posture conditions.

Several systematic reviews in the past decade have explored the impact of bolus consistency on swallowing and the effectiveness of thickened liquids as an intervention for impaired swallowing safety (5–9). Although these reviews used different article inclusion criteria and explored slightly different questions, they concur in concluding that thicker consistencies of liquid are less likely to be aspirated than thin liquids (5–7). Whether long-term use of thickened liquids leads to reductions in downstream negative sequelae such as pneumonia remains unclear (7–9).

One recent study with results that diverge from the systematic review evidence was reported by Miles and colleagues (10). Unlike the majority of studies considered in the systematic reviews, Miles and colleagues used Flexible Endoscopic Evaluations of Swallowing (FEES) to evaluate swallowing safety in a prospective sample of 180 in-patients referred for FEES assessments at two urban hospitals. Worst airway protection status was classified for each participant per exam, based on the depth of airway invasion and whether or not a cough response was seen with material visualized on or below the true vocal folds for 5 ml and 50 ml challenges of thin and mildly thick liquid. The authors reported that although the mildly thick liquid trials showed an overall reduction in the frequency of aspiration compared to thin liquids, some patients who showed a cough in response to airway invasion on thin liquids failed to show a cough response to airway invasion with a thicker consistency of the same volume.

The question of whether a thicker consistency is effective in reducing penetration or aspiration may sound deceptively straight forward, but a number of methodological issues can influence the answer to this question, both in research and in clinical practice. These include the number of boluses that are tested for each consistency, the order of bolus presentation, control of potentially confounding factors such as bolus volume, and the handling of varying scores across repeated boluses of the same consistency. In research, additional considerations that vary across studies with respect to methodological rigor include rater reliability and blinding of raters to patient, consistency, order of the bolus within the protocol, and audio information in the recording. The most common reporting practice, both in research and in clinical practice, is to summarize performance within a given patient or research participant based on the worst swallowing safety status seen across boluses of a particular consistency (11, 12). This practice ignores both the frequency and range in severity of penetration-aspiration events, inflating the contribution of single occurrences of more-serious airway invasion events (13, 14). Furthermore, although most studies collect data for each bolus using the 8-point Penetration-Aspiration Scale (PAS) (15), it is common for the full scale scoring details to be reduced to 2-, 3-, or 4- levels prior to analysis and reporting. Unfortunately, there is no consensus regarding scale reduction practices and differences in choices hinder meaningful comparisons being drawn across studies (11, 12).

One legitimate rationale for summarizing PAS scores for research analyses lies in prerequisite assumptions of common statistical tests. Traditional count-based approaches (e.g., chi-square, simple logistic regression) require that data for each participant are statistically independent. Therefore, these analyses are unable to include individual data points across repeated bolus trials of a given condition for a single participant, necessitating summarization (i.e., taking the maximum or modal score). In the presence of elevated within- participant variability [e.g. (16, 17),] the summarization of PAS scores may not adequately represent swallowing function. While some may ignore this statistical assumption, its violation is known to produce highly inaccurate effect size estimates (18). Moreover, these types of analyses are unable to account for missing data, which can be common in PAS datasets, particularly when safety-motivated procedural stopping criteria result in termination of testing for a given consistency prior to collection of all planned repeated trials. Overall, approaches that summarize repeated trials within a participant result in a loss of information and detail. This issue is particularly concerning in studies exploring the physiological mechanisms behind penetration and aspiration, where the assumption of similar physiology across both safe and unsafe swallows from an individual classified as either an aspirator or a non-aspirator is unlikely to be valid.

In the case of repeated boluses in a swallowing protocol, multilevel models (i.e., hierarchical or mixed effects models) are a potential alternative to resolve the issue of non-independence. These models rely on a partial pooling approach that accounts for the nested, non-independent structure of the data via random effects. Their ability to incorporate multiple trials for each participant not only increases statistical power and improves the accuracy of effect sizes, but also reduces the frequency of false positive findings (19, 20). One limiting factor of multilevel models is inability to achieve model convergence in a frequentist framework, which relies on maximum likelihood or restricted maximum likelihood methods for model fitting, due to small random effect variances or sample sizes. However, this issue can be easily resolved with a Bayesian framework by incorporating uncertainty (i.e., prior knowledge) before the data are analyzed. Given these benefits, Bayesian multilevel models have quickly become the standard analysis approach in many fields and offer richer analysis possibilities than traditional frequentist models (e.g., *t*-test, chi-square test, simple regression) across a wide range of outcomes.

In this paper, we use Bayesian multilevel ordinal regression models to examine patterns of airway invasion across different liquid consistencies. This is a secondary analysis of available bolus-level PAS data collected in three prior studies for which the second author served as the principal investigator. In contrast to previous published analyses of these datasets (either in full or for diagnostic subgroups within each project) (17, 21–26), the primary analysis approach taken in this paper allows for consideration of repeated bolus presentations in the swallowing protocol and does not collapse PAS score data into binary categories of safe and unsafe. Our goal was to answer the following research questions:

Q1: How do different liquid consistencies affect PAS scores?
Q2: Among participants with airway invasion (PAS > 2) on thin liquids, how do thicker liquid consistencies impact PAS scores?
Q3: Do PAS scores on thickened liquids differ between liquids thickened with a commercially available starch-based thickener vs. a commercially available xanthan gum-based thickener?
Q4: To enable comparison with the recent Miles et al. paper (10), how frequently do thickened liquids result in improvement or worsening based on summarized worst PAS scores?

## 2 Methods

### 2.1 Available data

The source data for the analyses in this manuscript came from the following projects:

(1) Two industry funded studies (henceforth referred to as Projects/Datasets 1a and 1b): Project 1a, in which videofluoroscopy data for repeated trials of thin, mildly thick, moderately thick and extremely thick liquids were obtained from adults at risk for non-congenital, nonsurgical, and non-oncologic oropharyngeal dysphagia. The study sample included those with stroke or other brain injury aged ≥ 20 years and other in-patients or out-patients aged ≥ 50 years with dysphagia risk (i.e., patient-reported symptoms justifying an assessment) (17). b) Project 1b, in which videofluoroscopy data for repeated trials of thin, mildly thick and moderately thick liquids were obtained from hospitalized in-patients at risk for dysphagia due to primary diagnoses of stroke, traumatic brain injury, Parkinson Disease, Multiple Sclerosis, Alzheimer Disease, other dementia, or other medically complex conditions with suspected dysphagia. (unpublished; ClinicalTrials.gov protocol ID NCT03387267).
(2) R01 funding from the National Institutes of Health to the second author for a project (henceforth referred to as Project/Dataset 2) to collect quantitative videofluoroscopic measures of swallowing across liquids of different consistencies, both in healthy adults and in cohorts of individuals with suspected dysphagia due to several medical diagnoses (Amyotrophic Lateral Sclerosis, Parkinson Disease, traumatic spinal cord injury, following radiation treatment for oropharyngeal cancer, and following COVID-19 infection) (17, 21–26). ClinicalTrials.gov protocol IDs NCT04114617, NCT05594173, NCT03192358, NCT04114604, NCT04112940 and NCT04537650).

**Figure 1:**
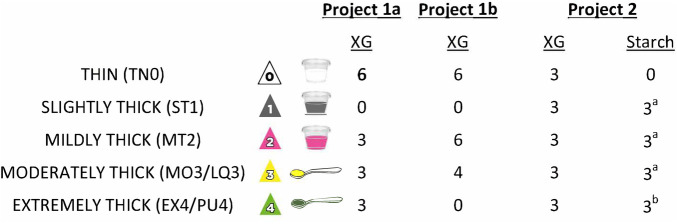
Details of the different bolus presentation protocols used across the three studies. XG = Xanthan Gum. ^a^included in some but not all of the patient cohorts; ^b^not included in the patient cohorts.

Certain elements of data collection were common to all three of the source projects, namely collection of videofluoroscopy data at 30 frames per second, with multiple boluses per consistency, beginning with blocks of thin liquid and progressing to thicker consistencies. All barium stimuli were prepared in 20% w/v concentration according to standard recipes. For Projects 1a and 1b, the barium sulfate product used was Varibar® Thin (Bracco Diagnostics). For the NIH dataset, the barium sulfate product used was E-Z-Paque® powdered barium (Bracco Diagnostics). Thicker consistencies were prepared by adding commercial thickening agents to the thin barium recipe; for the thicker liquids, consistency was confirmed as falling into Levels 1 to 4 of the International Dysphagia Diet Standardisation Initiative (IDDSI) Framework, using IDDSI's testing methods (27, https://www.iddsi.org). Henceforth, these consistencies will be referred to using IDDSI terminology and/or abbreviations: thin (TN0), slightly thick (ST1), mildly thick (MT2), moderately thick (MO3) and extremely thick (EX4). It should be noted that according to the IDDSI Framework, moderately thick (MO3) and extremely thick (EX4) liquids are identical in flow characteristics to liquidized (LQ3) and pureed foods (PU4); therefore, we will use the abbreviations MO3/LQ3 and EX4/PU4 for these consistencies. For all 3 projects, cups containing 40–60 ml of each test stimulus were arranged in blocks in a muffin tray in the order of presentation. For each trial, participants were instructed to pick up a new cup, to take a comfortable sip and to swallow naturally without waiting for a cue from the clinician. For the moderately and extremely thick stimuli, boluses were taken by teaspoon rather than sip. Where possible, participants self-administered the test boluses. Finally, pre- and post-sip cup weights were collected on a digital balance to enable derivation of sip weight and sip volume. For Project 2, thickened stimuli were prepared using two different thickeners: (a) Resource® ThickenUp® Clear™ (Nestlé Health Science), a xanthan-gum based thickening agent; and (b) Resource® ThickenUp® (Nestlé Health Science), a starch-based thickening agent. Figure 1 illustrates the different study protocols with respect to the stimulus consistencies tested, the thickeners used, and the number of boluses presented per consistency.

### 2.2 Data processing and PAS rating

All three projects shared the same core lab for videofluoroscopy rating. Data processing involved splicing the original videofluoroscopy recordings into shorter clips, each containing the swallowing events for a single bolus. At this point, the audio track was also removed and black screen scrubbers were superimposed on the recordings to blind raters to any potentially biasing information such as participant identifiers, bolus consistency labels, bolus number in the protocol and the video time code. Each bolus clip was then relabeled with a random file number, and assigned as part of a batch of ∼150 bolus clips to two trained raters who documented the number of swallows seen for each bolus, and PAS ratings for each swallow. Inter-rater agreement for PAS rating was inspected for each batch; discrepant ratings were flagged and sent to a consensus meeting for review and resolution. Each consensus meeting was attended by 2–3 members of the rating team, but these were not necessarily the same individuals who provided the original ratings for any given clip. Additional details regarding the rating procedures and rater reliability can be found in the supplemental materials published with the primary articles for Projects 1a and 2 (17, 21).

### 2.3 Statistical analysis

Due to the previously mentioned differences in study protocols, we divided this retrospective analysis into two parts. In part 1, we analyzed the combined data from datasets 1a and 1b. In part 2, we analyzed the data from dataset 2. We used Bayesian multilevel ordinal regression models with a logit-link to estimate differences in patterns of airway invasion across liquid consistencies. A Bayesian framework afforded several benefits, including flexibility to use maximal random effects structures, quantification of uncertainty, and ease of model interpretation. All models included random intercepts of participant, which permitted multiple trials for each participant (i.e., resolved violations of independence) and incomplete data (e.g., participants without trials of extremely thick consistency), as well as a random slope of consistency, which allowed each participant to have their own effect of consistency. Model predictions provided the probability of PAS scores for each fixed effect. To estimate a weighted PAS score across trials, these probabilities were multiplied by their respective PAS scores for each fixed effect and then summed (28).

Before proceeding with the analysis, we first inspected the data to answer two preliminary questions, which influenced choices regarding factors to include in the models. First, recognizing that multiple swallows could occur for a single bolus, we examined whether PAS scores meaningfully differed by swallow number. The results of this preliminary exploration can be found in Appendix A, and show that the probability of higher PAS scores did not meaningfully increase across repeated swallows for a single bolus. Therefore, the maximum PAS score across swallows was calculated for each bolus and used in subsequent analyses. Second, we wanted to determine whether PAS scores varied across diagnoses. Statistical models with and without a fixed effect of diagnosis were compared via leave-one out cross validation (28) for Q1 and Q2. The results of this exploration showed that diagnosis did not meaningfully contribute to the models (Table B1).

**Table B1:** Model comparisons via leave-one-out cross-validation for Q1.

Aim	Dataset	Model	ELPD difference	SE difference
Q1	1	With Diagnosis	0	0
		Without Diagnosis	−6.42	3.59
	2	With Diagnosis	0	0
		Without Diagnosis	−0.33	3.08
Q2	1	Without Diagnosis	0	0
		With Diagnosis	−1.07	2.40
	2	Without Diagnosis	0	0
		With Diagnosis	−2.85	1.70

ELPD, expected log pointwise predictive density; SE, standard error.

On this basis, the models for Q1 examining group-level differences in PAS scores across bolus consistencies included a fixed effect of consistency. For thin liquids, within-subject variability and saturation across repeated bolus trials were also described for each dataset. For Q2, we used the same models but limited the analysis to the subset of data for participants with at least one score of concern (PAS > 2) on thin liquids. Q3 examined how different thickening agents affected PAS scores and was limited to dataset 2. Here, we first compared models with and without a fixed effect of consistency via leave-one out cross validation. Results indicated that consistency did not meaningfully contribute to the model (Table B2). Thus, the final model included a fixed effect of thickening agent only and excluded thin liquid boluses.

**Table B2:** Model comparisons via leave-one-out cross-validation for Q3.

Aim	Dataset	Model	ELPD Difference	SE Difference
Q3	2	Without Consistency	0	0
		With Consistency	−2.96	4.67

ELPD, expected log pointwise predictive density; SE, standard error.

**Figure C1:**
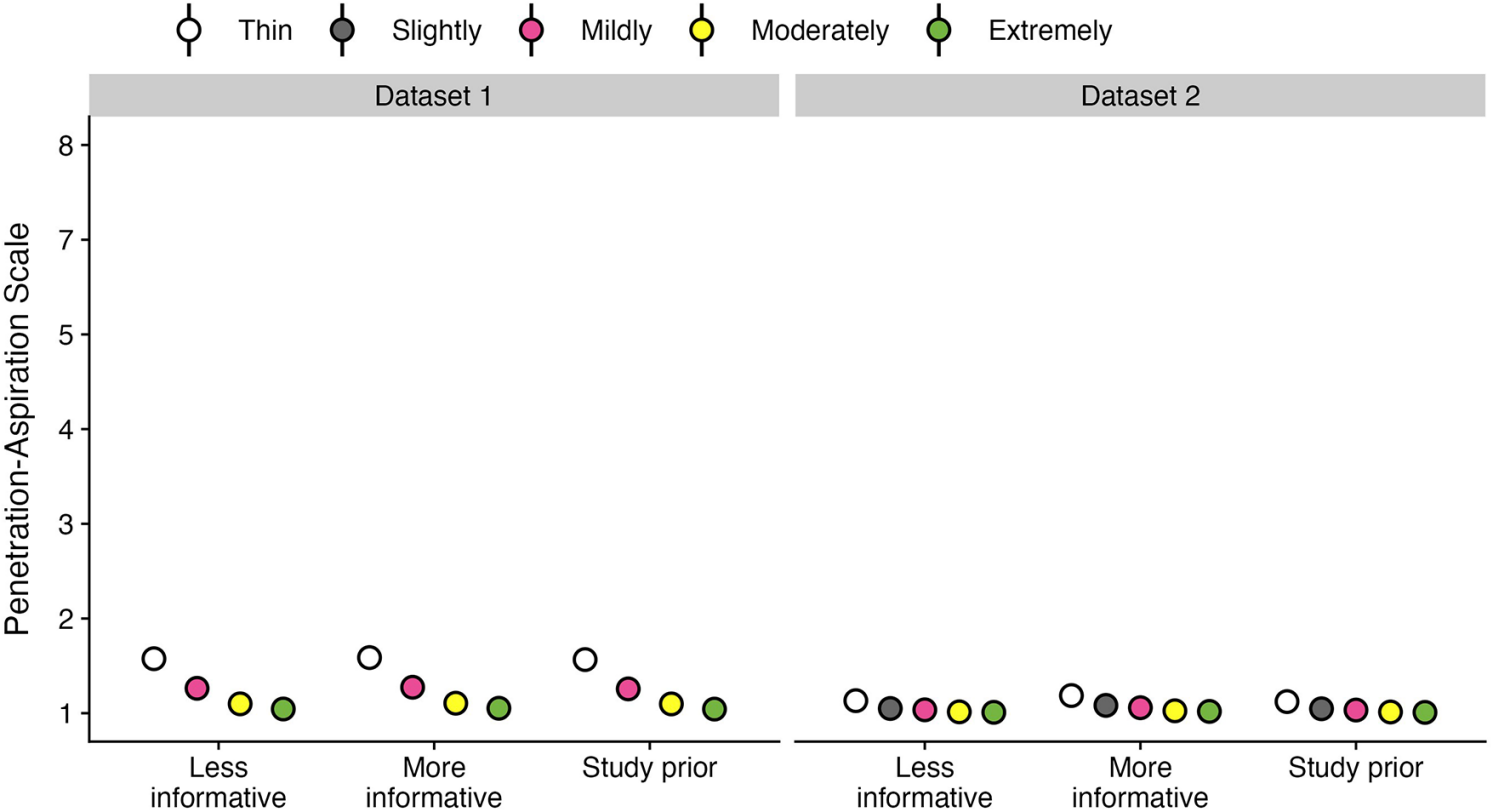
Q1 results of the prior sensitivity analysis.

**Figure C2:**
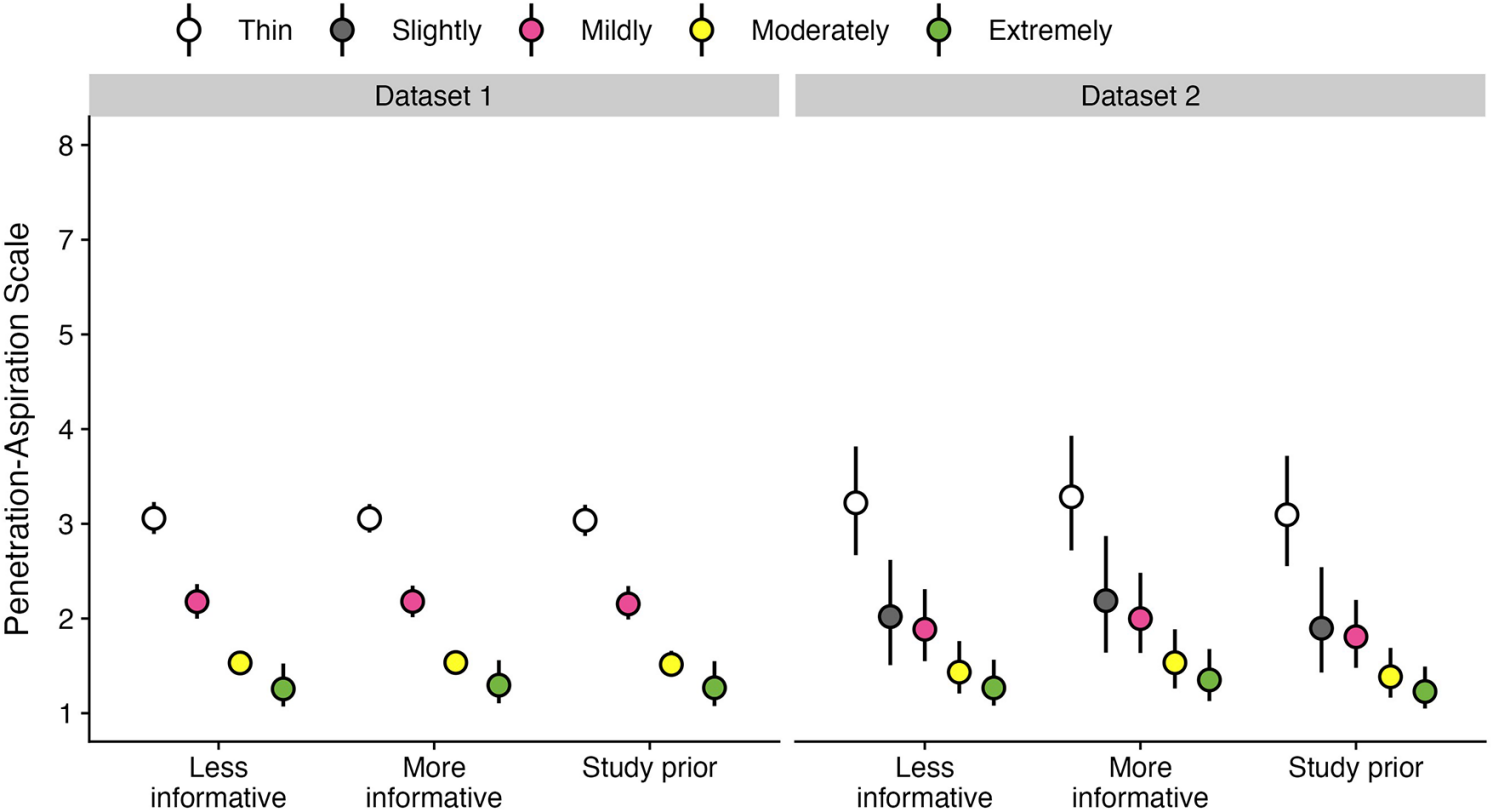
Q2 results of the prior sensitivity analysis.

**Figure C3:**
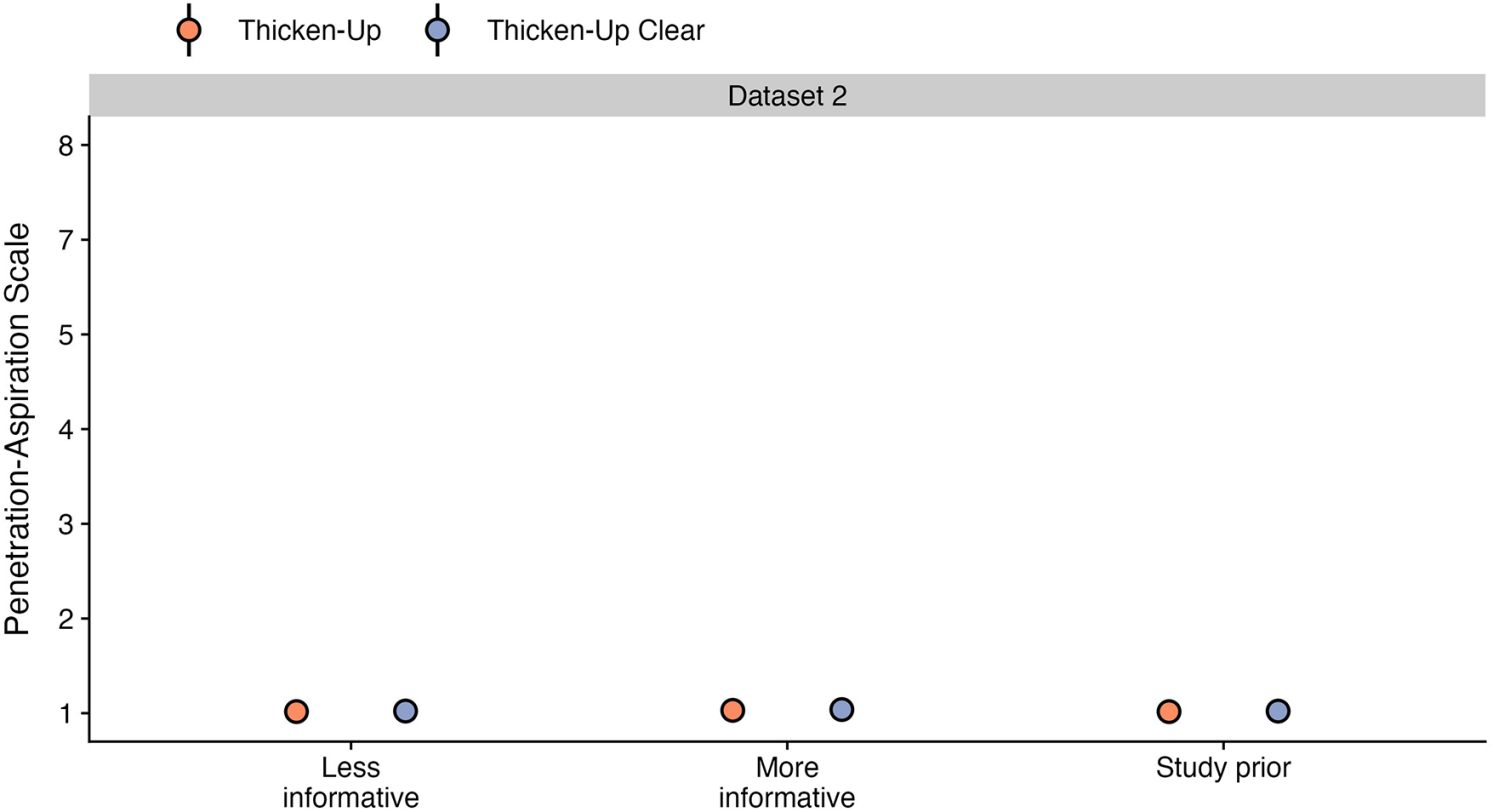
Q3 results of the prior sensitivity analysis.

Analyses were performed in R version 4.2.1 (R Core Team, 2018) with the *brms* package (29). Models used “weakly informative” prior distributions for fixed and random effects, which assumed no *a priori* effects were present and restricted implausible parameter values. All models demonstrated adequate convergence from sampling diagnostics. Post-hoc alternate prior specifications were performed, which revealed that model inferences remained stable regardless of the prior distribution (Figures C1–C3).

For Question 4, we identified the worst (i.e., highest) PAS score for each participant for thin liquids and across all thickened consistencies combined. These worst scores were cross-tabulated, with frequencies (and percentages) calculated at the participant level.

## 3 Results

### 3.1 Demographics

#### 3.1.1 Dataset 1

The combined data from project 1a and 1b comprised a total of 8,185 boluses from 678 participants (Table 1). The cohort included 389 males (57%) and 289 females (43%) with an average age of 68 years (SD = 16). Diagnoses included stroke (37%), healthy older adults (25%), unknown diagnosis (23%), Parkinson Disease (4%), other neurologic diagnoses (4%), acquired brain injury (3%), pneumonia (2%), dementia (1%), spinal cord injury (0.37%), and chronic obstructive pulmonary disease (0.18%). Since PAS scores of 6 were rarely appreciated (*n* = 9; 0.16%), this score was excluded from analyses with this dataset.

**Table 1:** Overall frequencies (percent) of penetration-aspiration scale scores by bolus consistency for Q1.

	Penetration-Aspiration scale								
	Consistency (IDDSI label)	1	2	3	4	5	6	7	8
Dataset 1 (*n* = 678)	Thin (TN0)	1,981 (60.2%)	651 (19.8%)	248 (7.5%)	28 (0.9%)	249 (7.6%)		43 (1.3%)	88 (2.7%)
	Mildly (MT2)	1,458 (68.5%)	330 (15.5%)	150 (7%)	10 (0.5%)	121 (5.7%)		11 (0.5%)	50 (2.3%)
	Moderately thick (MO3/LQ3)	1,623 (81.5%)	171 (8.6%)	102 (5.1%)	4 (0.2%)	76 (3.8%)		1 (0.1%)	15 (0.8%)
	Extremely thick (EX4/PU4)	730 (94.2%)	33 (4.3%)	6 (0.8%)	0 (0%)	4 (0.5%)		1 (0.1%)	1 (0.1%)
Dataset 2 (*n* = 177)	Thin (TN0)	412 (79.4%)	57 (11%)	18 (3.5%)	2 (0.4%)	20 (3.9%)	1 (0.2%)	3 (0.6%)	6 (1.2%)
	Slightly thick (ST1))	623 (85.7%)	65 (8.9%)	12 (1.7%)	1 (0.1%)	19 (2.6%)	1 (0.1%)	0 (0%)	6 (0.8%)
	Mildly thick (MT2)	693 (90.7%)	44 (5.8%)	15 (2%)	0 (0%)	10 (1.3%)	0 (0%)	0 (0%)	2 (0.3%)
	Moderately thick (MO3/LQ3)	650 (94.9%)	19 (2.8%)	6 (0.9%)	0 (0%)	9 (1.3%)	1 (0.1%)	0 (0%)	0 (0%)
	Extremely thick (EX4/PU4)	690 (96.9%)	15 (2.1%)	2 (0.3%)	1 (0.1%)	3 (0.4%)	1 (0.1%)	0 (0%)	0 (0%)

#### 3.1.2 Dataset 2

Dataset 2 comprised 3,407 boluses from 177 participants (Table 2). The cohort included 83 males (47%) and 94 females (53%) with an average age of 54 years (SD = 18). Diagnoses included healthy young adults (60%), Parkinson's disease (12%), post-COVID (12%), amyotrophic lateral sclerosis (11%), spinal cord injury (3%), and oropharyngeal cancer post-radiation (3%).

**Table 2:** Overall frequencies (percent) of penetration-aspiration scale scores by bolus consistency for Q2 (limited to participants who showed at least one PAS score > 2 on thin liquids).

	Penetration-Aspiration scale								
	Consistency (IDDSI label)	1	2	3	4	5	6	7	8
Dataset 1 (*n* = 299)	Thin (TN0)	391 (29.7%)	271 (20.6%)	248 (18.8%)	28 (2.1%)	249 (18.9%)		43 (3.3%)	88 (6.7%)
	Mildly (MT2)	451 (46.7%)	207 (21.5%)	138 (14.3%)	8 (0.8%)	109 (11.3%)		10 (1%)	42 (4.4%)
	Moderately thick (MO3/LQ3)	579 (66.9%)	110 (12.7%)	90 (10.4%)	3 (0.3%)	68 (7.9%)		1 (0.1%)	15 (1.7%)
	Extremely thick (EX4/PU4)	100 (86.2%)	12 (10.3%)	1 (0.9%)	0 (0%)	2 (1.7%)		0 (0%)	1 (0.9%)
Dataset 2 (*n* = 33)	Thin (TN0)	27 (29.7%)	14 (15.4%)	18 (19.8%)	2 (2.2%)	20 (22%)	1 (1.1%)	3 (3.3%)	6 (6.6%)
	Slightly thick (ST1))	73 (51.4%)	33 (23.2%)	12 (8.5%)	1 (0.7%)	16 (11.3%)	1 (0.7%)	0 (0%)	6 (4.2%)
	Mildly thick (MT2)	73 (60.3%)	23 (19%)	14 (11.6%)	0 (0%)	9 (7.4%)	0 (0%)	0 (0%)	2 (1.7%)
	Moderately thick (MO3/LQ3)	77 (75.5%)	10 (9.8%)	6 (5.9%)	0 (0%)	8 (7.8%)	1 (1%)	0 (0%)	0 (0%)
	Extremely thick (EX4/PU4)	72 (84.7%)	7 (8.2%)	2 (2.4%)	0 (0%)	3 (3.5%)	1 (1.2%)	0 (0%)	0 (0%)

### 3.2 Q1: How do different liquid consistencies affect PAS scores?

**Figure 2:**
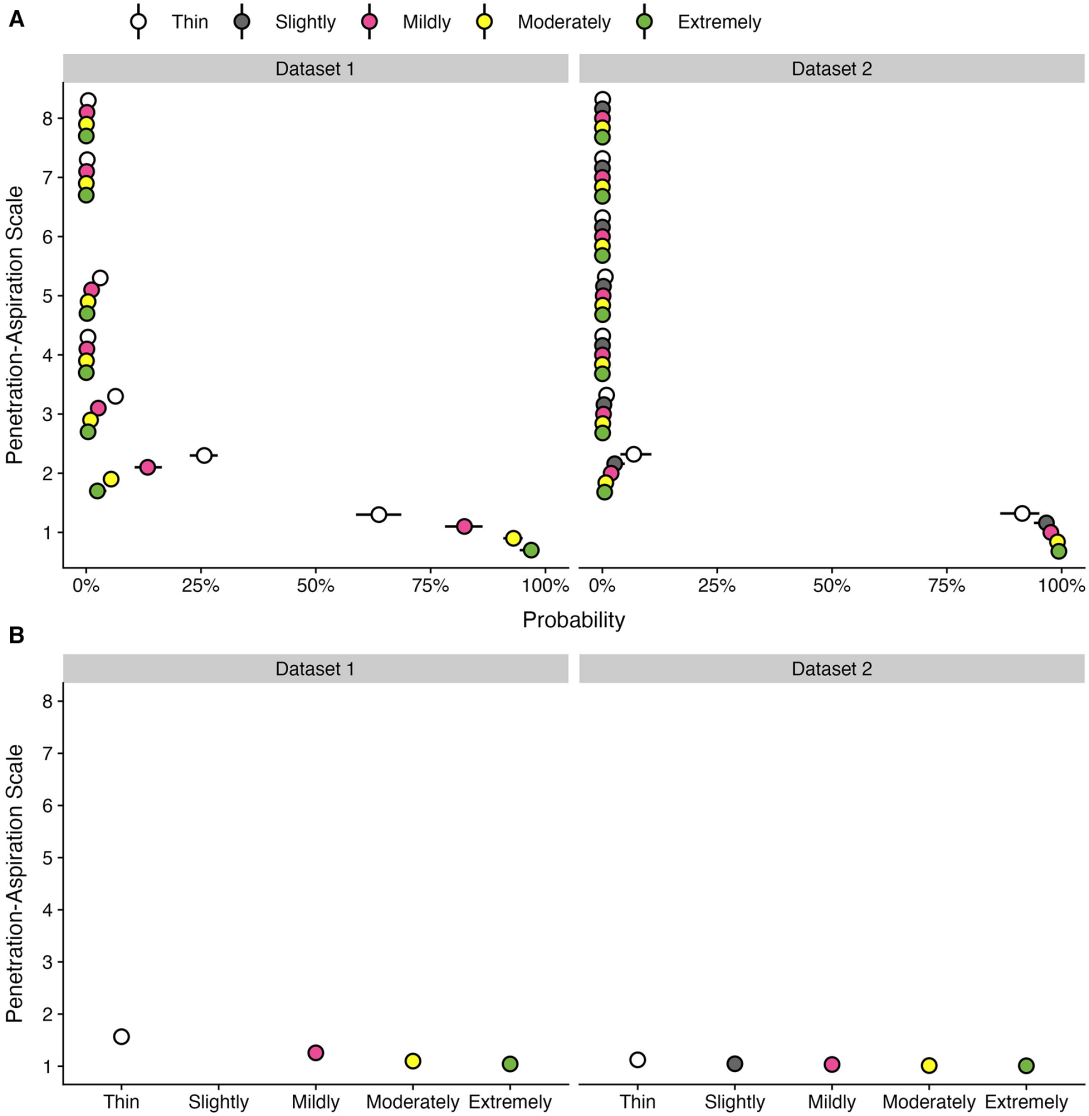
Effect of bolus consistencies on (**A**) the probability of PAS scores and (**B**) the weighted PAS score obtained from the model posterior distribution. Panel **A** displays the probability and corresponding 95% credible interval for each PAS score, stratified by bolus consistency across two datasets. Panel **B** displays the estimated posterior median average (i.e., weighted) PAS scores by bolus consistency with 95% credible intervals. This is estimated by multiplying each PAS score's numerical value (1–8) by its probability (**A**) and summing these weighted values (28). Note that all estimates were obtained from ordinal regression models.

Table 1 provides data regarding the frequency (number and percent) of boluses displaying different PAS scores by bolus consistency for each dataset. Figure 2 provides an illustration of the Q1 results for both datasets.

#### 3.2.1 Dataset 1

Of the 8,185 individual bolus datapoints in this dataset, 6,977 (85%) displayed PAS scores of 1 or 2. Penetration (i.e., PAS scores of 3, 4 or 5) was seen on 998 (12%) boluses and aspiration (i.e., PAS scores of 6, 7 or 8) was seen on 210 (3%) boluses. Across all boluses, the probability of a score of concern (PAS > 2) was higher for thin liquid boluses (10.55%; 95% CI: 8.32, 13.13) compared to mildly (4.25%; 95% CI: 3.08, 5.68), moderately (1.53%; 95% CI: 1.04, 2.11), and extremely thick (0.66%; 95% CI: 0.26, 1.23). These differences were reflected in the weighted PAS scores, such that thin liquids showed a PAS score of 1.57 (95% CI: 1.48, 1.66), mildly thick was 1.26 (95% CI: 1.2, 1.33), moderately thick was 1.1 (95% CI: 1.07, 1.13), and extremely thick was 1.04 (95% CI: 1.02, 1.08). Notably, the magnitude of differences between consistencies was small with several comparisons that were not meaningfully different, including mildly vs. moderately and moderately vs. extremely thick.

#### 3.2.2 Dataset 2

Of the 3,407 individual bolus datapoints in this dataset, 3,268 (96%) displayed PAS scores of 1 or 2. Penetration (i.e., PAS scores of 3, 4 or 5) was seen on 118 (3%) boluses and aspiration (i.e., PAS scores of 6, 7 or 8) was seen on only 21 (1%) boluses. This reflects the strong proportion of healthy adults in the dataset. Across all boluses, the probability of a score of concern (PAS > 2) was higher for thin liquid boluses (1.75%; 95% CI: 0.82, 3.06) compared to slightly (0.65%; 95% CI: 0.26, 1.3), mildly (0.46%; 95% CI: 0.2, 0.85), moderately (0.18%; 95% CI: 0.06, 0.37), and extremely thick (0.12%; 95% CI: 0.03, 0.27) consistencies. Weighted PAS scores showed differences across bolus consistencies. For example, thin liquids had higher PAS scores (1.12; 95% CI: 1.07, 1.19) compared to mildly (1.03; 95% CI: 1.02, 1.06), moderately (1.01; 95% CI: 1.01, 1.02), and extremely thick (1.01; 95% CI: 1, 1.02); however, there were no meaningful differences compared to slightly thick (1.05; 95% CI: 1.02, 1.09). Additionally, there were no meaningful differences between slightly and mildly thick consistencies, as well as between mildly, moderately, and extremely thick consistencies. 

### 3.3 Q2: Among participants with airway invasion (PAS > 2) on thin liquids, how do thicker liquid consistencies impact PAS scores?

**Figure 3:**
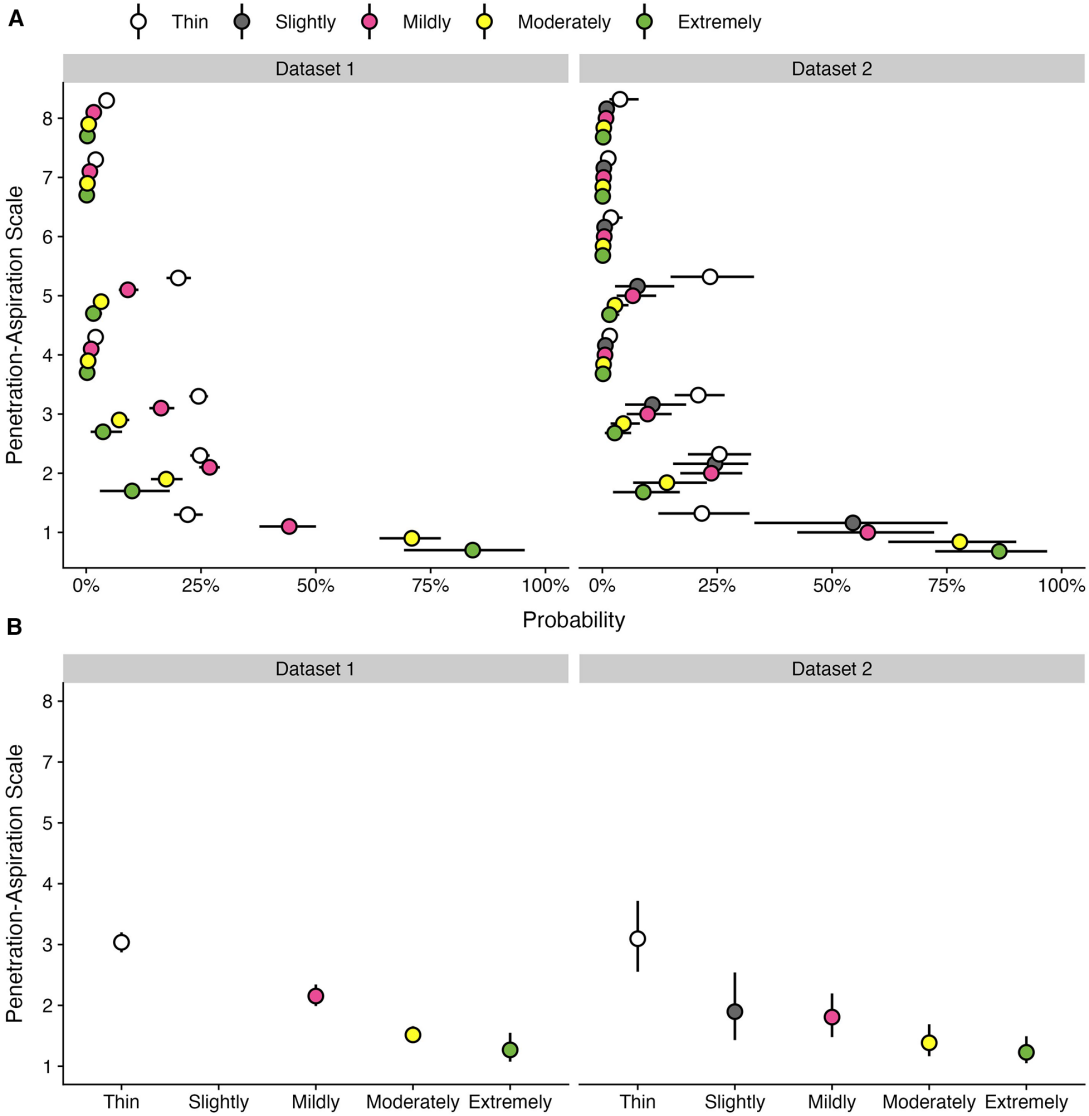
Effect of bolus consistencies on (**A**) the probability of PAS scores and (**B**) the weighted PAS score obtained from the model posterior distribution among participants with airway invasion on at least one thin liquid bolus. Panel **A** displays the probability and corresponding 95% credible interval for each PAS score, stratified by bolus consistency across two datasets. Panel **B** displays the estimated posterior median average (i.e., weighted) PAS scores by bolus consistency with 95% credible intervals. This is estimated by multiplying each PAS score's numerical value (1–8) by its probability (**A**) and summing these weighted values (28). Note that all estimates were obtained from ordinal regression models.

Table 2 provides data regarding the frequency (number and percent) of boluses displaying different PAS scores by bolus consistency for the subset of participants in each dataset who displayed at least one PAS score > 2 on thin liquids. Figure 3 provides an illustration of the results for Q2 across both datasets.

#### 3.3.1 Dataset 1

Two-hundred and ninety-nine participants (44.1%) demonstrated at least one score of concern (PAS > 2) on thin liquids. More than 74% of participants completed at least five bolus trials of thin liquid, while 35% of participants completed six bolus trials. Across repeated bolus trials of thin liquid, the first score of concern (i.e., first trial of PAS > 2) was most likely to be appreciated on the first thin liquid bolus trial (*n* = 155, 51.84%). The second (*n* = 69, 23.07%), third (*n* = 32, 10.70%), and fourth (*n* = 27, 9.03%) presentations of thin liquid showed slightly lower occurrences of the first PAS score of concern, whereas this was less commonly seen on the fifth (*n* = 11, 3.68%) and sixth (*n* = 5, 1.67%) trials.

Among participants with at least one trial of airway invasion on thin liquids, the probability of a score of concern (PAS > 2) was substantially higher for thin liquid boluses (53.11%; 95% CI: 46.43, 60.41) compared to mildly (28.9%; 95% CI: 23.34, 35.43), moderately (11.68%; 95% CI: 8.48, 15.57), and extremely thick (5.84%; 95% CI: 1.49, 12.61) consistencies. Weighted PAS scores differed across bolus consistencies, such that the average thin liquid PAS score was 3.04 (95% CI: 2.87, 3.2), mildly thick was 2.15 (95% CI: 1.99, 2.34), moderately thick was 1.51 (95% CI: 1.39, 1.66), and extremely thick was 1.27 (95% CI: 1.08, 1.55). The magnitude of these weighted PAS scores differed across consistencies, such that PAS scores decreased as bolus consistency increased; however, PAS scores for moderately and extremely thick consistencies were not meaningfully different.

Table 3 provides frequencies for single vs. multiple PAS scores of concern by consistency for each dataset. PAS scores of concern are further stratified into two degrees of severity (PAS > 2; PAS > 5). Among participants with at least one trial showing a PAS > 2 across all consistencies, multiple scores of concern across repeated trials were more commonly seen for thin liquids (62.22%) compared to mildly (49.47%), moderately (35.20%), and extremely thick (9%) consistencies. A similar pattern was appreciated among participants with at least one trial of aspiration (PAS > 5).

**Table 3:** Frequencies of single and repeated scores of concern stratified by dataset.

Consistency (IDDSI Label)	Number of scores of concern	Dataset 1		Dataset 2	
		PAS > 2	PAS > 5	PAS > 2	PAS > 5
		Freq (%)	Freq (%)	Freq (%)	Freq (%)
Thin (TN0)	1	112 (37.46%)	58 (63.74%)	20 (60.60%)	2 (33.33%)
	2	89 (29.77%)	28 (30.77%)	9 (27.27%)	4 (66.67%)
	3	45 (15.05%)	3 (3.30%)	4 (12.12%)	0 (0%)
	4	35 (11.71%)	2 (2.20%)	0 (0%)	0 (0%)
	5	17 (5.69%)	0 (0%)	0 (0%)	0 (0%)
	6	1 (0.33%)	0 (0%)	0 (0%)	0 (0%)
Slightly thick (ST1)	1	N/A	N/A	8 (50%)	1 (33.33%)
	2	N/A	N/A	0 (0%)	0 (0%)
	3	N/A	N/A	2 (12.50%)	2 (66.67%)
	4	N/A	N/A	5 (31.25%)	0 (0%)
	5	N/A	N/A	1 (6.25%)	0 (0%)
Mildly thick (MT2)	1	96 (50.53%)	42 (82.35%)	9 (52.94%)	2 (100%)
	2	49 (25.79%)	8 (15.69%)	6 (35.29%)	0 (0%)
	3	32 (16.84%)	1 (1.96%)	2 (11.76%)	0 (0%)
	4	13 (6.84%)	0 (0%)	0 (0%)	0 (0%)
Moderately thick (MO3/LQ3)	1	81 (64.80%)	13 (93%)	2 (28.57%)	1 (100%)
	2	25 (20%)	0 (0%)	2 (28.57%)	0 (0%)
	3	9 (7.20%)	1 (7%)	2 (28.57%)	0 (0%)
	4	10 (8%)	0 (0%)	1 (14.28%)	0 (0%)
Extremely Thick (EX4/PU4)	1	10 (91%)	2 (100%)	5 (83.33%)	1 (100%)
	2	1 (9%)	0 (0%)	1 (16.67%)	0 (0%)

Note that two different thresholds for “scores of concern” are provided: (1) airway invasion (PAS > 2) and (2) aspiration (PAS > 5).

N/A, not applicable.

#### 3.3.2 Dataset 2

Thirty-three participants (18.64%) demonstrated at least one score of concern (PAS > 2) on thin liquids. More than 94% of participants completed at least three bolus trials of thin liquid. Across repeated bolus trials of thin liquid, the first score of concern (i.e., first trial of PAS > 2) was most likely to be appreciated on the first thin liquid bolus trial (*n* = 21, 11.86%), whereas the second (*n* = 9, 5.14%) and third (*n* = 3, 1.80%) presentations showed lower occurrences of the first score of concern.

Among participants with at least one trial of airway invasion on thin liquids, the probability of a score of concern (PAS > 2) was higher for thin liquid boluses (52.89%; 95% CI: 33.41, 78.28) compared to slightly (20.92%; 95% CI: 8.23, 39.9), mildly (18.5%; 95% CI: 8.99, 31.44), moderately (8.14%; 95% CI: 3, 15.94), and extremely thick (4.69%; 95% CI: 0.03, 0.27) consistencies. Weighted PAS scores also differed across bolus consistencies, such that there were higher PAS scores on thin liquids (3.1; 95% CI: 2.55, 3.72) compared to slightly (1.9; 95% CI: 1.43, 2.54), mildly (1.81; 95% CI: 1.48, 2.2), moderately (1.39; 95% CI: 1.16, 1.69), and extremely thick (1.23; 95% CI: 1.05, 1.49) consistencies. Notably, there were no meaningful differences in the magnitude of weighted PAS scores between slightly vs. mildly, mildly vs. moderately, and moderately vs. extremely thick. 

The frequencies of single vs. multiple PAS scores of concern, stratified by degree of severity (PAS > 2; PAS > 5) are shown on the right hand side of Table 3 by consistency. Among participants with at least one trial showing a PAS > 2 across all consistencies, multiple scores of concern across repeated trials were more commonly seen for moderately thick liquids (71.43%), although there was a low number of airway invasion events in this dataset due to the large proportion of healthy participants.

### 3.4 Q3: Do PAS scores on thickened liquids differ between liquids thickened with a commercially available starch-based thickener vs. a commercially available xanthan gum based thickener?

#### 3.4.1 Dataset 2

**Figure 4:**
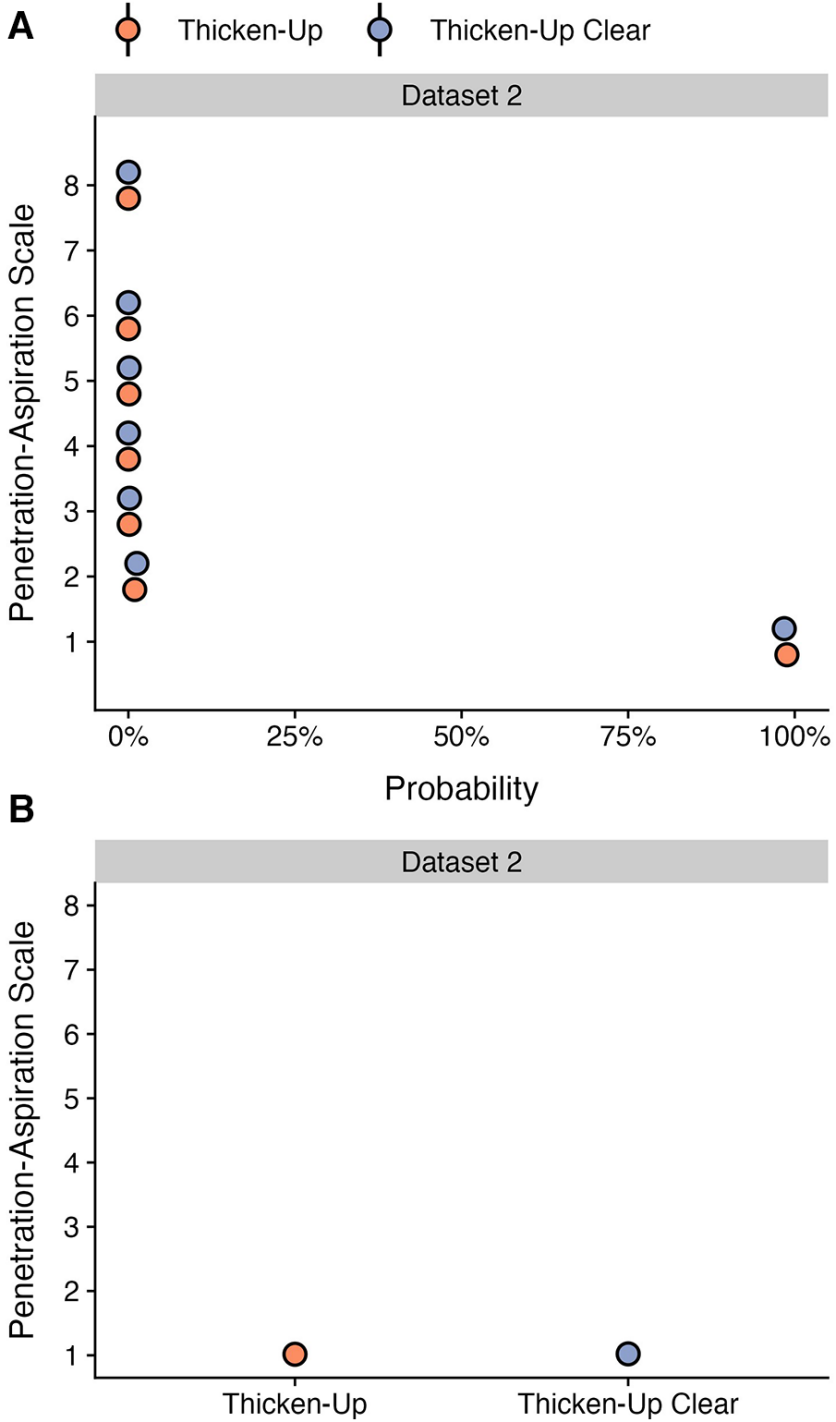
Effect of different thickening agents on (**A**) the probability of PAS scores and (**B**) the weighted PAS score obtained from the model posterior distribution. Panel **A** displays the probability and corresponding 95% credible interval for each PAS score, stratified by thickening agent. Panel **B** displays the estimated posterior median average (i.e., weighted) PAS scores by thickening agent with 95% credible intervals. This is estimated by multiplying each PAS score's numerical value (1–8) by its probability (**A**) and summing these weighted values (28). Note that all estimates were obtained from ordinal regression models and that these estimates are averaged across consistencies.

This question was only explored in dataset 2, for which both starch and xanthan gum-based thickeners were used. Stimuli prepared with both thickeners were tested for all consistencies in 78 of the healthy participants, while selected thicknesses were included in the protocols for specific clinical cohorts, resulting in available data for a total of 125 participants. Table 4 shows the frequencies (percent) of different PAS scores by thickener, across all bolus consistencies. As shown in Figure 4 panel A, participants demonstrated similar probabilities for scores of concern (PAS > 2) between stimuli prepared with Thicken-Up (0.23%; 95% CI: 0.08, 0.47) and Thicken-Up Clear (0.31%; 95% CI: 0.11, 0.62). As shown in Figure 4 panel B, weighted PAS scores were also nearly identical across the two thickeners: Thicken-Up (1.016; 95% CI: 1.007, 1.029) and Thicken-Up Clear (1.022; 95% CI: 1.01, 1.039).

**Table 4:** Distribution of penetration-aspiration scale scores for Q3 (dataset 2 only).

	Penetration-Aspiration scale									
	Consistency (IDDSI label)	Thickener	1	2	3	4	5	6	7	8
Aim 3 (*n* = 125)	Slightly thick (ST1)	TU	308 (87.5%)	28 (8%)	7 (2%)	1 (0.3%)	5 (1.4%)	0 (0%)	0 (0%)	3 (0.9%)
		TUC	315 (84.7%)	37 (9.9%)	5 (1.3%)	0 (0%)	12 (3.2%)	1 (0.3%)	0 (0%)	2 (0.5%)
	Mildly thick (MT2)	TU	242 (93.1%)	13 (5%)	3 (1.2%)	0 (0%)	2 (0.8%)	0 (0%)	0 (0%)	0 (0%)
		TUC	331 (89%)	22 (5.9%)	10 (2.7%)	0 (0%)	7 (1.9%)	0 (0%)	0 (0%)	2 (0.5%)
	Moderately thick (M03/LQ3)	TU	318 (95.2%)	9 (2.7%)	4 (1.2%)	0 (0%)	2 (0.6%)	1 (0.3%)	0 (0%)	0 (0%)
		TUC	332 (94.6%)	10 (2.8%)	2 (0.6%)	0 (0%)	7 (2%)	0 (0%)	0 (0%)	0 (0%)
	Extremely thick (EX4/PU4)	TU	226 (99.1%)	1 (0.4%)	1 (0.4%)	0 (0%)	0 (0%)	0 (0%)	0 (0%)	0 (0%)
		TUC	338 (95.5%)	11 (3.1%)	1 (0.3%)	1 (0.3%)	2 (0.6%)	1 (0.3%)	0 (0%)	0 (0%)

Sample size at the participant-level (*n* = 125) is shown in the first column, whereas values in the body of the table indicate the frequency and percentage of Penetration-Aspiration Scale scores at the trial-level.

TU, thicken-up (starch-based thickener); TUC, thicken-up clear (xanthan gum based thickener).

### 3.4 How frequently do thickened liquids result in improvement or worsening based on summarized worst PAS scores?

**Figure 5:**
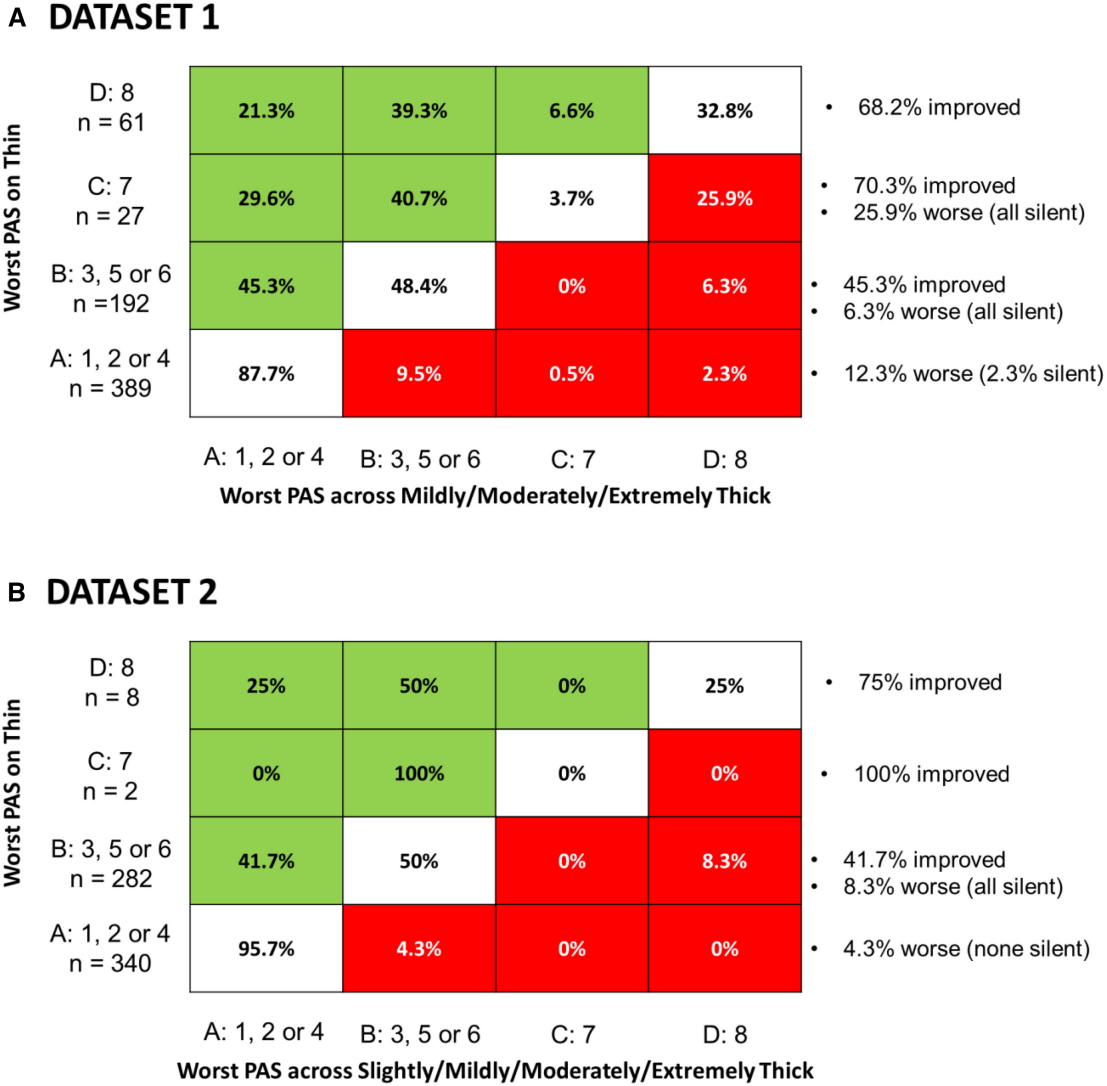
Cross-tabulation of the proportion of participant-worst PAS score categories for across polled thicker consistencies by participant-worst thin liquid PAS score category. Each cell represents the percent of participants with a worst PAS category of (A–D) on thin liquids (*y*-axis) for which a worst PAS category of (A–D) was seen across all of the thicker consistencies combined (*x*-axis). Green shading is used to indicate score improvement with thicker consistencies while red sharing indicates score worsening, Annotations on the right side of the table for each row summarize the frequencies of improvement or worsening seen by worst PAS score category on thin liquids. Panel **A** shows results for dataset 1 while Panel **B** shows equivalent results for dataset 2.

This question was explored to enable comparison with the recent paper by Miles et al. (10), which reported worsening of aspiration from sensate on thin liquids to silent on thickened liquids in some patients. Figure 5 shows the crosstabulation of participant-worst scores for thin liquids and across all of the thickened consistencies combined. Panel A shows the results for dataset 1 and panel B for dataset 2. PAS scores are summarized categorically as suggested by Steele & Grace-Martin (11), with scores of 1, 2 and 4 grouped together (Category A) as scores either showing no bolus entry into the laryngeal vestibule or scores showing transient penetration with complete ejection of material back out of the airway. Category B includes PAS scores of 3, 5 and 6 in which there is penetration but material remains in the laryngeal vestibule at the end of the swallow. PAS scores of 7 and 8 are captured in categories C and D, respectively, with category D (PAS of 8) reflecting cases of silent aspiration. The cells in the Figure 5 tables represent the percent of cases with a worst PAS category of A, B, C or D on thin liquids (y-axis) for which a worst PAS category of A, B, C or D was seen across all of the thicker consistencies combined (x-axis). Green shading is used to indicate score improvement with thicker consistencies while red sharing indicates score worsening. Annotations on the right side of the table for each row summarize the frequencies of improvement or worsening seen by worst PAS score category on thin liquids. The reader can appreciate that although the frequencies of worse scores are not zero, worsening was rare in both datasets and the overwhelmingly dominant trend is towards improvement with thickened liquids. The one notable exception to this statement is seen in datasetdataset 1 for the 27 participants (4%) who displayed a worst PAS score of 7 on thin liquids. Of these, 7 participants (25.9%) showed worsening, with at least one event of silent aspiration across the combined thicker liquid trials.

## 4 Discussion

This manuscript involves secondary analysis of two large datasets of videofluoroscopy data, collected across three prior projects. In total, the two datasets comprised bolus-level PAS ratings for 8,185 boluses (from 678 participants) and 3,407 boluses (from 177 participants), respectively. Although slightly different bolus protocols were used across the three studies, as illustrated in Figure 1, the rating procedures for both datasets were identical and performed in the same central analysis lab. All boluses were rated by two trained raters who were blinded to each other's ratings and to potentially biasing information including participant identity, diagnosis, sex and age, bolus consistency, and bolus trial order within the testing protocol. All discrepancies in PAS scores were flagged and resolved by consensus. As such, a high degree of rigor was used in the rating procedures for all three prior studies. The analyses performed for this manuscript involved pooling of data from projects 1a and 1b, which shared the use of Varibar® barium sulfate and identical recipes for thickening to mildly thick or thicker consistencies using the same commercially available xanthan gum thickener. Analysis of dataset 2 was kept separate due to use of a different barium product, the inclusion of slightly thick liquids, and the availability of data for both starch and xanthan gum thickened liquids. Additionally, dataset 2 involved a substantial proportion of healthy participants, in whom penetration-aspiration events of concern would be unexpected.

Unlike previous analyses of these datasets, and other datasets in the literature, the statistical analysis used to address questions 1–3 in this manuscript allowed for consideration of all boluses across repeated trials of each consistency, and avoided reduction of the Penetration-Aspiration Scale. The results are generally concordant with previous findings synthesized across studies in systematic reviews, namely showing significant reductions in the frequency and severity of penetration-aspiration with liquids thickened to mildly thick consistency or thicker, compared to thin liquids. However, in contrast to prior work, these results are more trustworthy due to the inclusion of multiple bolus trials that account for within-participant variability with a Bayesian multilevel modeling approach. Given substantial sample sizes in both datasets, results were highly stable and robust regardless of the choice of prior distribution—an important and reassuring validation of these findings (see Appendix C).

In question 4, we adopted the traditional practice of summarizing worst PAS scores per participant for thin vs. thicker liquid consistencies to enable comparison to the results reported by Miles et al. (10). It should be noted that there are a number of differences in data collection methods between our studies and the Miles et al. study, including the use of videofluoroscopy rather than FEES, different bolus volumes, different stimuli (non-barium liquids), differences in the number of trials per condition, and different categorization of PAS score severity. Like those reported by Miles and colleagues, our data do show that a small proportion of participants showed worse airway invasion (i.e., higher PAS scores) on thicker liquids. Of particular note are the 7 participants whose worst scores moved from PAS = 7 (i.e., aspiration followed by an unsuccessful sensate clearing attempt) to PAS = 8 (i.e., silent aspiration). This finding emphasizes the fact that cough responses to aspiration may not be effective for ejecting material out of the airway, and that PAS scores of 7 indicate severe impairments of airway protection similar to scores of 8. However, the predominant trend in these datasets was one of improved airway protection with thickened liquids. It should be noted that this trend was seen even with summarization of worst scores across a larger number of thickened liquid trials (i.e., a minimum of 9 in dataset 1 and a minimum of 12 in the dataset 2) compared to the number of thin liquid trials in the data collection protocols (i.e., 6 and 3 in datasets 1 and 2, respectively).

As with any research study, limitations must be acknowledged for the analyses reported in this manuscript. These include the lack of balanced sample sizes for different clinical diagnostic subgroups in the datasets (although the exploration of diagnosis in Appendix B found no meaningful differences). Additionally, we were unable to explore the impact of bolus volume on penetration-aspiration due to the use of comfortable, patient-selected sip volumes in the studies from which the data were sourced. Prior analyses of those studies have reported descriptive statistics for sip-volume based on pre- and post-swallow cup weights (13, 17, 21). Importantly, all three source projects involve the acknowledged confound that the moderately and extremely thick liquids were taken using a teaspoon, which limited bolus volume to the 4–6 ml range rather than the ∼ ≥ 10 ml volumes seen for the thin, slightly and mildly thick consistencies that were sipped from a cup. Thus, part of the observed effect of thickening to moderately and extremely thick consistencies on swallowing safety may be attributable to smaller bolus volumes. Another methodological constraint of the studies explored in this manuscript is the fact that thicker liquids were always tested later in the data collection protocol. Thus, order effects, either with respect to improved airway protection with practice or with respect to possible worsening of airway protection with fatigue cannot be ruled out. Additionally, it is acknowledged that all of the data collected in the source studies required the use of a low concentration of barium sulfate contrast agent, which differs from regular liquids in taste.

## 5 Conclusion

The secondary analyses described in this manuscript provide strong confirmatory evidence that penetration-aspiration is much less likely to occur on thicker liquid consistencies than with thin liquids. The evidence in this paper corroborates previous findings that swallowing safety may vary within an individual across repeated trials of a given consistency, such that multiple trials are needed to rule out penetration-aspiration both with thin liquids (13, 30), and with thicker consistencies. Additionally, the findings show that a small number of patients may show worse airway protection with thicker consistencies, supporting best practice guidance that thicker consistencies should not be recommended without both prior assessment and subsequent monitoring for adverse outcomes.

## Figures and Tables

**Figure A1:**
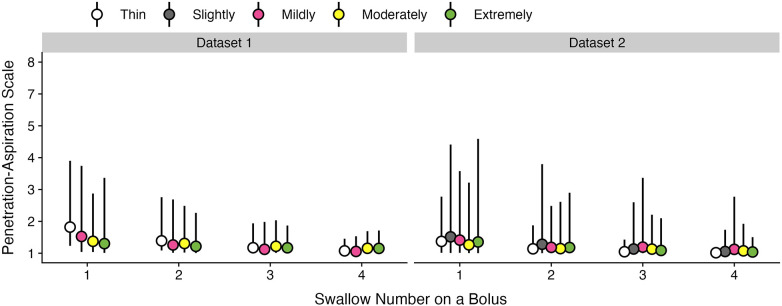
Effect of swallow number on weighted penetration-aspiration scale scores across consistencies. Estimated posterior median average (i.e., weighted) PAS scores are shown with 95% credible intervals, stratified by swallow number and bolus consistency. This estimate is derived by multiplying each PAS score's numerical value (1–8) by its probability and summing these weighted values (28). Note that all estimates were obtained from ordinal regression models.

**Figure A2:**
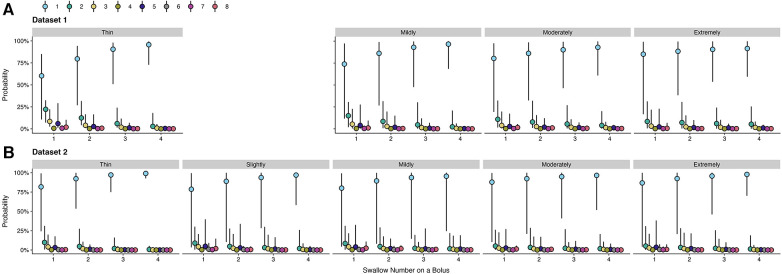
Probabilities of penetration-aspiration scale scores across swallow number and consistencies. The probability of each PAS score and corresponding 95% credible intervals are displayed, stratified by swallow number and consistency.

## Data Availability

The data analyzed in this study is subject to the following licenses/restrictions: The datasets generated during and/or analyzed during the current study are not publicly available due to ethical/Legal restrictions. Requests to access these datasets should be directed to catriona.steele@uhn.ca.

## Appendix A: analysis of the effect of swallow number on penetration-aspiration scale scores

Since different Penetration-Aspiration Scale (PAS) scores can occur across multiple swallows within a given bolus trial, this was first measured at the swallow-level. To determine whether PAS scores are meaningfully different across swallows, we performed an initial supplemental analysis to motivate subsequent analysis decisions. The statistical model included fixed effects of swallow number, consistency, and their two-way interaction, as well as a random slope of swallow number and random intercept of participant. Below we provide a description of data from both datasets and visualization of model results.

### Results

For dataset 1, single swallows for a bolus accounted for 77.04% of the data, with a second swallow was seen in a further 17.54% of bolus trials. Three (3.93%) and four (1.1%) swallows per bolus were less common. The occurrence of five or more swallows per bolus was exceptionally rare (0.38%); therefore, only data from one to four swallows were included in the analysis.

For dataset 2, single swallows (75.72%) and two (18.07%) swallows per bolus were the most common patterns, whereas three (4.85%) and four (1.36%) swallows were less common. No participants had more than four swallows on a bolus.

Across both datasets, the first swallow on thin liquids showed higher weighted PAS scores than subsequent swallows (Figure A1); however, the magnitude of these differences was small. In dataset 1, for example, the weighted PAS score on the first thin liquid swallow was 1.85 (95% CI: 1.23, 3.58) compared to a PAS score of 1.41 (95% CI: 1.08, 2.56) on the second swallow. In dataset 2, the weighted PAS score on the first thin liquid swallow was 1.59 (95% CI: 1.01, 5.33) compared to a PAS score of 1.29 (95% CI: 1.00, 3.61) on the second swallow. All other bolus consistencies did not demonstrate meaningful differences across swallows. Figure A2 illustrates the probabilities for different PAS scores, with 95% credible intervals, at the swallow level.

## Appendix B: Supplemental description of statistical analyses

### Description of Bayesian prior distributions

*“*Weakly informative” prior distributions were used, such that they restricted implausible effects and did not assume that an *a priori* effect was present. For each aim, we assigned these prior distributions *a priori* for fixed and random effects. Random effects were prescribed an exponential distribution with a standard deviation of one. This distribution ensured that random effects were positive with 95% of the probability density below a standard deviation of four. For fixed effects, a normal distribution centered at zero with a standard deviation of five was used, which provided a wide 95% probability density ranging from log-odds of −10 to 10.

### Model comparison results

Models are ordered by best-fit as determined by leave-one-out cross-validation (31). ELPD difference is the difference in the expected log pointwise predictive density for two models. SE difference is the standard error of the difference. To determine which model provided optimal fit, ELPD difference is divided by SE difference. The inclusion of either diagnosis or consistency did not demonstrate elpd_diff/se_diff > |2|, which is a common threshold for leave-one-out cross-validation (31). Therefore, these variables were not included, and the most parsimonious model was reported.

## Appendix C: Prior sensitivity analysis

Here we determined whether inferences remained stable with alternate prior specifications. After data analysis was completed, additional models with either more or less informative priors were fit and results from these models were compared. In this study, our prior distribution was a normal distribution centered at zero with a standard deviation of five. Across all models, we fit two additional prior distributions that were either “more” or “less” informative. A normal distribution centered at zero with a standard deviation of 2 was used for our alternate “more informative” prior distribution, which further constrained parameter values. A flat (i.e., uniform) prior was used to represent a “less informative” prior distribution. This prior distribution was less informative such that all parameter values were equally probable, which is equivalent to a frequentist analysis.

These models were performed for each aim and a weighted PAS score was calculated across bolus consistencies with its associated 95% credible interval. This is estimated by multiplying each PAS score's numerical value (1–8) by its probability from the statistical model and then summing these weighted values (28).

Across all aims, results indicated that model inferences remained robust despite different prior distributions. Furthermore, the magnitude of model estimates and 95% credible intervals remained nearly identical.
